# Environmental tobacco smoke exposure and health disparities: 8-year longitudinal findings from a large cohort of Thai adults

**DOI:** 10.1186/s12889-015-2547-y

**Published:** 2015-12-08

**Authors:** Thanh Tam Tran, Vasoontara Yiengprugsawan, Dujrudee Chinwong, Sam-ang Seubsman, Adrian Sleigh

**Affiliations:** Research School of Population Health, College of Medicine, Biology and Environment, The Australian National University, Building 62, Mills Rd, Acton 2601, Canberra, Australia; Faculty of Pharmacy, Chiang Mai University, Chiang Mai, Thailand; School of Human Ecology, Sukhothai Thammathirat Open University, Nonthaburi, Thailand

**Keywords:** Environmental tobacco smoke, Thailand, Cohort study, Psychological distress, Wellbeing, Health-related quality of life

## Abstract

**Background:**

In rich countries, smokers, active or passive, often belong to disadvantaged groups. Less is known of tobacco patterns in the developing world. Hence, we seek out to investigate mental and physical health consequences of smoke exposure as well as tobacco-related inequality in transitional middle-income Thailand.

**Methods:**

We studied a nationwide cohort of 87,151 middle-aged and older adults that we have been following for eight years (2005–2013) for emerging chronic diseases. Logistic regression was used to identify attributes associated with passive smoke exposure. Longitudinal associations between smoke exposure and wellbeing (SF-8) or psychological distress (Kessler 6) were investigated with multiple linear regression or multivariate logistic regression analysis.

**Results:**

A high proportion of cohort members, especially females, were passive smokers at home and at public transport stations; males were more exposed at workplace and recreational places. We observed a social gradient with more passive smoking in poorer people. We also observed a dose response relationship linking graded smoke exposures (current, former, passive, non-exposed) to less wellbeing and more psychological distress (p-trend < 0.001). Female smokers in general had less wellbeing and more distress.

**Conclusion:**

Our findings add to current knowledge on the impact of active and passive smoking on health in a transitional economy. Promotion of smoking cessation programs both in public and at home could also potentially reduce adverse disparities in health and wellbeing in middle and lower income settings such as Thailand.

## Background

Active or passive, smoking kills and there is no safe level of exposure to it [1, 2]. The World Health Organization has estimated that tobacco kills nearly 6 million people each year and, among them, 10 % were due to second hand smoke [3, 4]. It is well-established that active smoking is associated with many deadly diseases, including diabetes, cardiovascular and respiratory illnesses, as well as lung and other cancers [3]. Smokers also have vastly reduced health-related quality of life [5–7] and more mental illness [8, 9]. Evidence against passive smoking is also compelling and there is a clear scientific consensus that environmental tobacco smoke can cause serious and fatal diseases in non-smoking adults and children [10].

In developing countries, information on passive smoking is scarce. Some developing countries have responded to the challenge of tobacco control and much can be learnt from their experience. Thailand is such a country and is well-known for its pioneering tobacco control policies that have been progressively implemented since 1992 when the parliament passed the Tobacco Products Control Act, banning advertising, promotion and sponsorship as well as sale to minors. By 2005, the Ministry of Health had banned display of cigarette at point of sale and had high impact horror pictures on all cigarette packages. In 2007, hotel lobbies, pubs and bars were declared non-smoking areas [11].

Despite the gains made since 1992, social disparities in smoking rates among Thais remain. For example, people belonging to low income or educational groups have highest smoking rates and those with highest income or educational levels have lowest smoking rates [12]. The laws limiting environmental smoke in 2007 are not well enforced [11]. Accordingly, there is still a major problem and little information about the effect of passive smoking. Given the high rate of smoking among those in socioeconomically disadvantaged groups, it seems likely that non-smokers of those same groups are disproportionately exposed to passive smoking. Passive smoking is involuntary and thus is an issue of equity and justice.

Gender disparity in passive smoking is also a known issue especially in patriarchal societies where gender differences in power may lead to women unwillingly but unavoidably exposed to environmental tobacco smoke (ETS) from male smokers [10]. Female passive smokers have also been shown to have worse health outcomes from ETS than male counterparts. They also reported more respiratory symptoms and worse self-rated health [13].

In this study, we investigate the effects of passive smoking on psychological distress and wellbeing in Thailand. Study participants are members of a large nationwide cohort that has been followed for a decade for research on changing disease patterns. Our study is one of the first to focus on environmental smoke and its effect on mental health, wellbeing and quality of life. It is also one of the first studies of ETS in a middle income Asian setting.

## Methods

### Study population and data collection

This report is part of an over-arching Thai Cohort Study (TCS) project that analyses the health-risk transition underway in Thailand. This transition involves changing health risks and outcomes of the Thai population as the country moves on from its traditional problems with infectious diseases and maternal-child mortality to emerging chronic conditions and injury. Participants of the TCS included 87,151 distance learning community-embedded students of Sukhothai Thammathirat Open University in 2005. Self-reported mail-out questionnaires with free return postage collected data at three time points: baseline 2005, follow-up 2009, and follow-up 2013. For this report, we use information from the first (2005) and the latest follow-up (2013).

Cohort characteristics have been reported extensively: in brief, TCS members represented well the adult Thai population for median age, average income, geographical distribution, religion and ethnic diversity [14–16]. Compared to the general population there was a small excess of females (54.3 %), and of urban dwellers (51.8 % vs 31.1 %). Cohort members are better educated than the general population and are ahead of their Thai compatriots on the health-risk transition path, representing aspirational “Thais of tomorrow”.

### Exposures, outcomes and confounders

TCS questionnaires collect data on a wide range of topics including demographic, socioeconomic and geographic information, health status, disease history, health-risk behaviours, health service use, social links and support, injuries, and family background.

Participants reported their smoking status (*never smoker*, *former smoker* or *current smoker*) at baseline (2005) and again at 4- and 8-year follow-ups (2009, 2013). At baseline in 2005, participants were also asked if they were exposed to smoke *at home*, in a r*ecreational place*, in the *workplace*, at a *public transport station* (e.g. train/bus station) or any *other place*. If they answered “yes” to any one of these locations they were classified as a *passive smoker*.

**Fig. 1:**
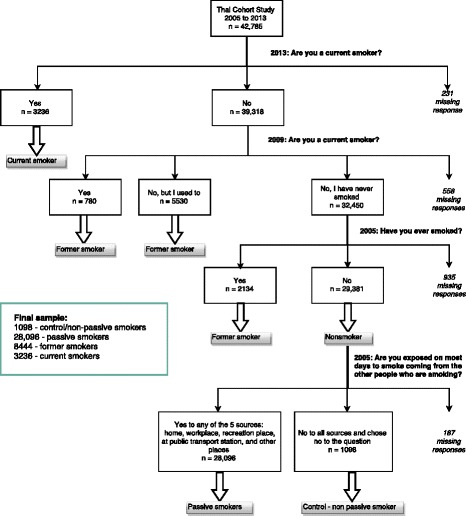
Selection of the analysed population from the Thai Cohort Study, 2005–2013

For analysis, participants were further classified into 4 different smoking groups as follows: 1) *control group* (non-exposure) – consistent never smokers on all three assessments who reported no exposure to passive smoke at baseline; 2) *passive smokers* – consistent never smokers in all three assessments who reported exposure to passive smoke at baseline; 3) *former smoker* (non-smoker in 2013 and smokers in at least one of the two previous assessments); 4) *current smoker* (in 2013 regardless of smoking status in 2009 or 2005) (Fig. 1).

Wellbeing was assessed in 2013 (i.e. at the end of the 8-year follow-up) using standardised Medical Outcome Study Short-Form 8 (MOS SF-8™) Health Survey [17, 18]. The original Short Form instrument (SF-36) is highly responsive to active smoking and is a sensitive marker for smoking related conditions [5, 7, 19]. SF-8 consists of eight questions representing the following eight domains: general health, physical functioning, daily physical, bodily pain, vitality, social functioning, mental health, and daily emotional [17]. Responses were on an ordinal 5 or 6 point scale. Physical Component Summary (PCS) score and Mental Component Summary (MCS) score were computed according to the SF-8 guidelines by first assigning international weights to each domain value before the domain scores were summed and a constant was then added. The scores were standardised using a norm-based scoring methods for a normal population with a mean score of 50 and standard deviation of 10. Higher scores represent better health outcomes.

Psychological distress was also measured in 2013 using standard Kessler 6 (K6) which has high validity for grading anxiety-mood disorders [20]. K6 consists of six questions “In the past 4 weeks, how often did you feel: 1) so sad nothing could cheer you up; 2) nervous; 3) restless or fidgety; 4) hopeless; 5) everything was an effort; and 6) worthless. Each question of this instrument uses a 5-category Likert response scale. Participants were given a score of 1 if answering “none”, 2 “a little”, 3 “some”, 4 “most”, and 5 for answering “all of the time”. The scores for all the questions were then totalled ranging from 6 to 30 with higher scores representing worse health outcomes. We combined the standard moderate and serious psychological distress categories to create a dichotomous outcome (<14: *no distress*, ≥14: *psychological distress*).

Information was also gathered on an array of covariates that could be potential confounders of smoking effects. These included demographic factors such as sex, age (grouped into 4 categories: <30, 30–39, 40–49, and ≥50), marital status (single or married/living with a partner), urbanisation status (classified into 4 groups based on urban (U) or rural (R) residency at age 10–12 and in 2005 (i.e. current) leading to lifecourse ruralites, urbanizers, de-urbanizers and urbanites [21].

Socioeconomic status (SES) was included in the analyses through three proxies: personal monthly income—very low (≤7000 baht), low (7001 to 10,000), middle (10,001 to 20,000), high (20,000 to 30,000), and very high (≥30,000 baht); educational attainment (high school, vocational study (diploma or certificate), university degree (bachelor or higher)); and household asset values (low (≤30,000 baht), medium (30,001 to 60,000), and high (>60,000)). In 2005, one US dollar equalled 42 Baht.

Other factors included for analysis were drinking and the total number of chronic health conditions reported. Participants were asked if they have ever drunk alcohol. Those who answered “No, never” were then considered *non-drinkers*; if they reported “used to drink before but now stopped”, they were classified as former drinkers. Other participants who responded either “occasional social drinker” or “current regular drinker” were classified as *alcohol drinkers*.

Participants were also asked to report whether they have been told by a doctor that they have one of the following conditions: diabetes (needing insulin or not), high cholesterol, high blood pressure, ischemic heart disease, stroke, cancers (liver, lung, digestive system, breast or other), goiter/thyroid abnormality, epilepsy, liver disease, chronic kidney disease, depression/anxiety, arthritis, pneumonia, chronic bronchitis, asthma, malaria, dengue fever, tuberculosis, other chronic infection, or any other diseases. Responses for all the health conditions were summed and grouped into 3 categories by number of illnesses: 0, 1, or ≥2.

Body size was assessed by Body Mass Index (BMI) in kg/m^2^, calculated as weight over height squared. Asian categories were used as follows: <18.5—underweight, ≥18.5 to <23—normal, ≥23 to <25—overweight at risk, ≥25 to <30—obese I, ≥30—obese II.

### Statistical analysis

Sociodemographic characteristics of different smoker categories were compared using bivariate frequency distributions. Similarly, the characteristics of passive smokers who reported different sources of ETS exposure were also examined.

To study the effects of smoking exposure on longitudinal wellbeing, we performed a multivariable linear regression with SF-8 summary scores at the 2013 endpoint as the outcome and 2005 baseline smoking status as the predictor. Subsequently, we performed a similar logistic regression with 2013 Kessler psychological distress as the dichotomous outcome. Finally, to study the effects of ETS exposure sources on wellbeing and psychological distress, we carried out another set of regressions (linear for SF8 as outcome and logistic for K6) with ETS exposure as predictor, restricted to passive smokers only.

All differences between means and proportions were tested by analysis of variance and the chi-square test, respectively. All p-values were 2-tailed with significance level set at 5 %. When important for analyses, test for linear trends were conducted and p-trend values produced. All statistical analyses were performed using STATA/SE 12.1 [22].

### Ethics approval

Informed written consent was obtained from all participants. All students were advised that they could withdraw, or not participate, without any effect on their academic progress. The questionnaires never sought sensitive personal information and no biological samples were taken. Ethics approval was obtained from Sukhothai Thammathirat Open University Research and Development Institute (protocol 0522/10) and the Australian National University Human Research Ethics Committee (protocols 2004/344 and 2009/570).

## Results

### Characteristics of non-smokers (non-passive vs passive) and smokers (former vs current)

Among the 40,874 participants analysed, only 2.7 % were never smokers with no exposure to passive smoke; the majority (66.8 %) were passive smokers (never smokers exposed to ETS) (Table 1). Approximately 21 % were former smokers and only 8 % reported smoking in 2013.

**Table 1:** Distribution of smoking status across socio-demographic attributes in 2005^a^

Attributes	Smoking status								Total (N)
	Non-exposure		Passive smoker		Former smoker		Current smoker		
	n	Row %	n	Row %	n	Row %	n	Row %	
Overall	1,098	2.7	28,096	68.7	8,444	20.7	3,236	7.9	40,874
Age (yrs)									
< 30	368	2.1	13,847	78.7	2,298	13.1	1,088	6.2	17,601
30–39	444	3.0	9,732	65.4	3,358	22.6	1,357	9.1	14,891
40–49	215	3.1	3,958	56.4	2,175	31.0	674	9.6	7,022
50+	71	5.2	559	41.1	613	45.1	117	8.6	1,360
Sex									
Male	273	1.5	7,951	42.7	7,325	39.3	3,095	16.6	18,644
Female	825	3.7	20,145	90.6	1,119	5.0	141	0.6	22,230
Marital status									
Single	490	2.5	15,418	77.1	2,805	14.0	1,285	6.4	19,998
Partnered	587	2.9	12,190	60.8	5,397	26.9	1,861	9.3	20,035
Urbanisation									
Rural-Rural	446	2.4	12,821	70.0	3,620	19.8	1,431	7.8	18,318
Rural–urban	304	2.6	7,923	67.2	2,678	22.7	881	7.5	11,786
Urban–rural	45	2.5	1,144	64.0	421	23.6	177	9.9	1,787
Urban-Urban	283	3.3	5,942	70.0	1,610	18.9	694	8.1	8,529
Personal income (Baht/month)									
Up to 3000	83	2.4	2,497	72.4	525	15.2	346	10.0	3,451
3000–7000	267	2.5	8,294	76.3	1,597	14.7	716	6.6	10,874
7000–10,000	185	2.0	6,499	70.7	1,776	19.3	739	8.0	9,199
10,000–20,000	303	2.7	7,242	63.5	2,884	25.3	973	8.5	11,402
> 20,000	233	4.5	3,009	58.1	1,535	29.6	401	7.7	5,178
Education									
High school	437	2.4	11,154	62.4	4403	24.6	1,887	10.6	17,881
Vocation	272	2.5	7940	72.8	1,897	17.4	798	7.3	10,907
University	387	3.2	8,936	74.5	2,127	17.7	545	4.5	11,995
Asset value									
Low	292	1.9	10,513	70.4	2,789	18.7	1,348	9.0	14,942
Middle	340	2.6	8,894	68.6	2,734	21.1	1,002	7.7	12,970
High	461	3.6	8,576	67.0	2,890	22.6	873	6.8	12,800
Drinking									
Non-drinker	579	5.1	10,268	91.1	297	2.6	129	1.1	11,273
Former drinker	61	1.7	1,841	52.5	1,265	36.1	339	9.7	3,506
Current drinker	456	1.8	15,903	61.3	6,834	26.3	2,743	10.6	25,936
No. of health condition									
0	469	3.2	10,270	70.7	2,604	17.9	1,191	8.2	14,534
1	501	2.6	13,747	70.0	3,941	20.1	1,443	7.4	19,632
≥ 2	127	1.9	4,042	61.0	1,871	28.2	592	8.9	6,632
Body size (BMI)									
Underweight	139	2.7	4,431	85.2	450	8.6	183	3.5	5,203
Normal	602	2.8	15,762	73.5	3,614	16.8	1,477	6.9	21,455
At risk	176	2.6	3,863	57.5	1,955	29.1	726	10.8	6,720
Obese I	134	2.3	3,066	52.0	2,007	34.1	688	11.7	5,895
Obese II	30	2.8	661	60.8	284	26.1	113	10.4	1,088

^a^analysis restricted to cohort members assessed in 2005, 2009 and 2013. Reflecting the large sample size, all variables significantly associated with smoking status

There were clear differences in smoking status between age groups and sexes (Table 1). Older age groups were more likely to be active smokers but less likely to be exposed to passive smoke. Most active smokers (95.6 %) were males and a majority of passive smokers (79 %) were females. The prevalence of smoking was slightly higher among married or partnered TCS members (8.9 %) compared to members who were single (6.2 %). There was no distinct pattern between urbanisation and smoking status.

Socioeconomic status (SES) was also highly correlated to ETS exposure, positively for increasing educational attainment and negatively for rise in income or assets. There was no substantial difference in proportion of current smokers between different income levels, but education and assets both showed that higher SES groups had lower active smoking rates.

Alcohol drinkers were more likely to be active smokers and less likely to be passive smokers. Meanwhile, a higher proportion of passive smokers were observed among the healthy non-smokers (with no or only 1 health condition). Former and current smokers were more common among those with multiple health conditions. There was no noticeable difference in the distribution of body size in the non-exposure group. Passive smokers were more likely to be underweight while former and current smokers had higher prevalence of overweight at risk and obesity class I.

### Location of exposure to ETS and characteristics of those exposed

For non-smokers who reported exposure to passive smoke, we tabulated the place of smoke exposure for different demographic and socioeconomic attributes (Table 2). Overall, only age, assets, income and alcohol drinking demonstrated notable differences within groups but these differences were still small (4 % or less). The most notable finding was the limited variation in passive smoking across the range of values within each location.

**Table 2:** Location of ETS exposure among passive smokers by cohort member attributes in 2005

Passive smokers	Total		Location of ETS exposure									
			At home		Recreational places		Workplace		Transport station		Other places	
	N	%^a^	n	%^a^	n	%^a^	n	%^a^	n	%^a^	n	%^a^
Overall	28,096	96.2	6,798	24.2	5,929	21.1	1,173	4.2	15,285	54.4	14,863	52.9
Age												
< 30	13,847	97.4	4,033	29.1	3,258	23.5	5,272	38.1	8,016	58.9	7,192	51.9
30–39	9,732	95.6	2,032	20.9	1,895	19.5	4,116	42.3	5,043	51.8	5,146	52.9
40–49	3,958	94.9	652	16.5	690	17.4	1,612	40.7	1,955	49.4	2,209	55.8
≥ 50	559	88.7	81	14.5	86	15.4	173	31.0	271	48.5	316	56.5
Gender												
Male	7,951	96.7	1,247	15.7	2,036	25.6	4,259	53.6	3,925	49.4	4,152	52.2
Female	20,145	96.1	5,551	27.6	3,893	19.3	6,914	34.3	11,360	56.4	10,711	53.2
Marital status												
Single	15,418	96.9	3,865	25.1	3,545	23.0	5,953	38.6	8,929	49.9	8,270	53.6
Partnered	12,190	95.4	2,841	23.3	2,291	18.8	5,015	41.1	6,083	57.9	6,373	52.3
Urbanisation												
Rural-Rural	12,821	96.6	3,657	28.5	2,466	19.2	4,998	39.0	6,861	53.5	6,684	52.1
Rural–urban	7,923	96.3	1,503	19.0	1,735	21.9	3,391	42.8	4,468	56.4	4,192	52.9
Urban–rural	1144	96.2	279	24.4	258	22.6	441	38.6	654	57.2	591	52.7
Urban-Urban	5,942	95.5	1,314	22.1	1,429	24.1	2,235	37.6	3,156	53.1	3,285	55.3
Education												
High school	11,154	96.2	3,034	27.2	2,300	20.6	4,442	39.8	5,619	50.4	6,113	54.8
Diploma	7,940	96.7	2,104	26.5	1,605	20.2	3,307	41.7	4,367	55.0	4,016	50.6
University	8,936	95.8	1,641	18.4	2,004	22.4	3,406	38.1	5,260	58.9	4,699	52.6
Income												
Very low	2,497	96.8	937	37.5	634	25.4	471	18.9	1,439	57.6	1,515	60.7
Low	8,294	96.9	2,613	31.5	1,685	20.3	3,341	40.3	4,476	54.0	4,334	52.3
Middle	6,499	97.2	1,468	22.6	1,421	21.9	2,895	44.6	3,670	56.5	3,307	50.9
High	7242	96.0	1245	17.2	1457	20.1	3267	45.1	3892	53.7	3,737	51.6
Very high	3009	92.8	395	13.1	594	19.7	1116	37.1	1505	50.0	1640	54.5
Asset values												
Low	10,513	97.3	3,132	29.8	2,170	20.6	4,257	40.5	5,826	55.4	5,478	52.1
Middle	8,894	96.3	2,088	23.5	1,872	21.1	3,655	41.1	4,870	54.8	4,699	52.8
High	8,576	94.9	1,553	18.1	1,861	21.7	3,216	37.5	4,522	52.7	4,638	54.1
Drinking status												
Non-drinker	10268	94.7	2713	26.4	1,929	18.8	3,458	33.7	5611	54.7	5,326	51.9
Former	1841	96.8	439	23.9	437	23.7	734	39.9	1,039	56.4	976	53.0
Current	15903	97.2	3629	22.8	3,552	22.3	6,948	43.7	8,593	54.0	8,516	53.6
No. of health condition												
0	10270	95.6	2426	23.6	2,048	19.9	3,914	38.1	5413	52.7	5,161	50.2
1	13747	96.5	3351	24.4	2,869	20.9	5,489	39.9	7,489	54.5	7,362	53.6
≥ 2	4042	96.9	1014	25.1	1000	24.8	1,748	43.3	2358	58.4	2,326	57.6

All variables significantly associated with ETS location, except for gender and other places, assets and recreational places or other places, health condition and at home

^a^% = proportion of passive smokers reporting exposure to ETS at a specific location in 2005

All age groups reported transport stations as the most common source of passive smoke (approx. 50 %). Proportionately more females than males reported exposure to passive smoke at home and in public transport stations. Work and recreational places of exposure had higher proportions of male than female passive smokers. For males, workplaces were the most common source of exposure to passive smoke. Females were more commonly exposed at public transport stations.

Single cohort members were more likely than married peopled to be exposed to passive smoke at home, in recreational areas and public transport stations. The reverse is true for workplaces, with married people were more vulnerable to passive smoke. SES showed different patterns for different exposure places. All three measures agreed that lower SES non-smokers were more likely to be exposed to smoke at home; the trends were monotonic for increasing SES and decreasing reported exposure (Table 2).

### Smoke exposure, 8-year longitudinal wellbeing, and psychological distress

**Fig. 2:**
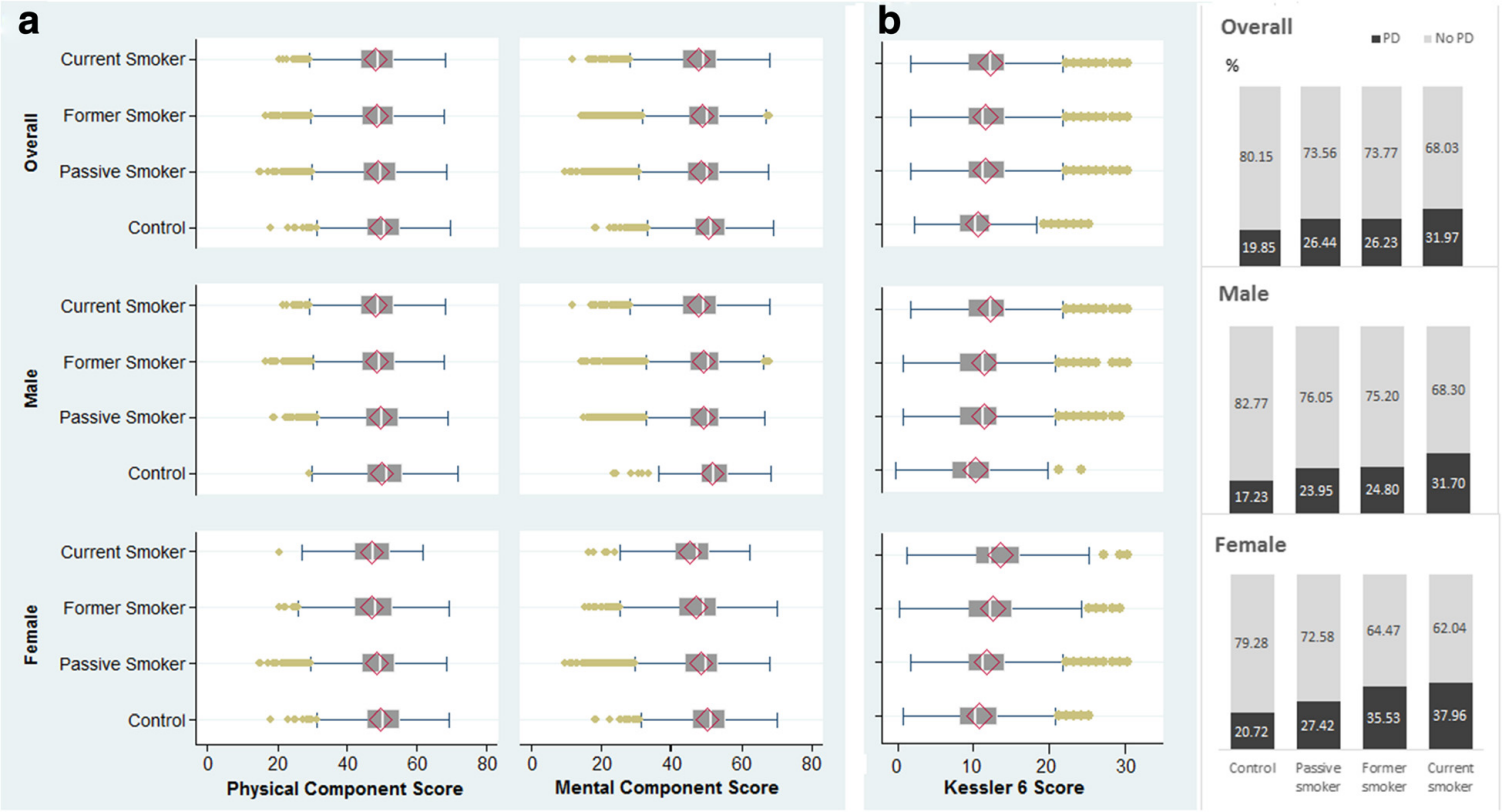
(**a**) SF-8 physical and mental component scores (PCS and MCS) and (**b**) Kessler 6 psychological distress scores and prevalence in 2013 by smoker status

For Kessler testing, in 2013 psychological distress affected 26.8 %; about 4 % of the cohort had serious psychological distress. For SF-8, mean scores were below 50 with physical component score (PCS) of 48.42, and mental component score (MCS) of 48.57 (Fig. 2).

Smoking status in 2005 correlated strongly with both SF-8 and Kessler 6 in 2013. In other 2005–2013 longitudinal analysis, non- smokers (i.e. ‘controls’) had the highest well-being score for both mental and physical components of the SF-8 (Fig. 2); they also have the lowest prevalence of psychological distress as found by Kessler 6. In general, 2005 passive smokers and former smokers were rather similar for their 2013 outcome scores. All smoker groups were similar for physical wellbeing. But mental wellbeing of current smokers was a problem as revealed in 2013 by low MCS and high prevalence of psychological distress.

As there was significant interaction between sex and smoking status, we present some 2013 analyses separately for men and women. For current smokers and controls, women had lower physical SF-8 scores than men (47.05 and 49.43 vs 48.12 and 49.84, respectively) and lower mental SF-8 scores (45.11 and 50.08 vs 47.69 and 51.47, respectively). Similarly, SF-8 scores for other categories of smoking (passive and former) also were worse among the females. Females also had worse Kessler scores: in 2013 the prevalence of psychological distress for controls and current smokers were 20.4 % and 36.9 % in females and 17.0 % and 31.0 % in males (Fig. 2).

To explore the effects of smoking on physical and mental wellbeing, we performed two regression tests: (1) linear regression with SF-8 PCS and MCS as 2013 outcomes; (2) logistic regression with dichotomous psychological distress as the 2013 outcome. Both models adjusted for 2005 baseline levels of demographic attributes (age groups, marital status, urbanisation), SES (personal income, household assets value, educational attainment) and other personal factors including drinking and numbers of health conditions. All these variables were previously shown in our bivariate analysis (and in literature reviewed [5–9]) to be associated with both smoking risk and poor mental health and wellbeing.

Baseline smoking status related inversely to longitudinal wellbeing outcome (Table 3). Wellbeing was highest for the non-exposure group and decreased progressively across smoker categories—passive, former and current. The monotonic trend linking smoking and wellbeing across smoking categories had larger increments and more significance for the mental component than the physical component (p-trends <0.001).

**Table 3:** Baseline smoking status and 2013 8-year longitudinal wellbeing and psychological distress^a^

Smoking status^b^		Wellbeing scores (SF-8)^c^				Psychological Distress (Kessler 6)^d^	
		Physical Component		Mental Component			
		aMD^e^	95 % CI	aMD^e^	95 % CI	aOR^f^	95 % CI
Overall	Control	Ref.		Ref.		1.00	.
	Passive smoker	−0.94***	−1.37 to −0.51	−1.29***	−1.78 to −0.80	1.29***	1.10 to 1.52
	Former smoker	−1.46***	−1.93 to −1.00	−1.67***	−2.19 to −1.14	1.48***	1.25 to 1.76
	Current smoker	−1.68***	−2.18 to −1.17	−2.71***	−3.28 to −2.14	1.87***	1.55 to 2.24
	*p-trend*	<0.001		<0.001		<0.001	
Male	Control	Ref.		Ref.		1.00	.
	Passive smoker	−0.44	−1.28 to 0.39	−1.75***	−2.68 to −0.81	1.26	0.90 to 1.76
	Former smoker	−0.91*	−1.75 to −0.06	−1.90***	−2.84 to −0.96	1.38	0.99 to 1.95
	Current smoker	−1.14**	−2.01 to −0.28	−2.90***	−3.87 to −1.94	1.76**	1.24 to 2.48
	*p-trend*	<0.001		<0.001		<0.001	
Female	Control	Ref.		Ref.		1.00	.
	Passive smoker	−1.13***	−1.64 to −0.62	−1.14***	−1.72 to −0.56	1.32**	1.09 to 1.59
	Former smoker	−2.12***	−2.78 to −1.45	−2.06***	−2.82 to −1.31	1.72***	1.37 to 2.16
	Current smoker	−2.15**	−3.46 to −0.85	−4.19***	−5.67 to −2.71	2.01**	1.33 to 3.02
	*p-trend*	<0.001		<0.001		<0.001	

*p < 0.05 **p < 0.01 ***p < 0.001

^a^outcomes adjusted for demographic attributes (age group, sex, marital status, urbanisation), SES (assets, income, education), and other factors (drinking, number of health conditions, body sizes)

^b^smoking status:—control (not active nor passive smokers from 2005 to 2013)

- passive smoker (not active but exposed to ETS in 2005)

- former smoker (previous smokers, 2005–2013)

- current smoker (active smoker in 2013)

^c^multiple linear regression for SF-8 physical and mental component scores

^d^binary logistic regression for psychological distress (Kessler 6 14–30) vs non distress (Kessler 6 6–13)

^e^adjusted mean difference

^f^adjusted odds ratio

Psychological distress in 2013 also displayed a strong monotonic trend relationship (p-trend < 0.001) with baseline cigarette smoke exposure in 2005 (Table 3). Compared to the non-exposure group, other smoking categories had higher relative odds of psychological distress after 8 years, as follows: passive (1.29), former (1.48) and current (1.87).

Sexes differed significantly for overall wellbeing or distress. Female smokers reported worse health than their male counterparts. Males exposed to passive smoke in 2005 did not have significantly lower PCS scores, whereas females did so. Former and current male smokers reported a PCS difference of approximately 1 from the control non-exposure group. In contrast, females estimated a PCS score difference of 2 comparing similar groups.

In contrast, passive smoking appeared to affect male’s mental wellbeing more than females. Exposure to any form of tobacco smoke (passive or active) significantly increased the odds of developing psychological distress in females. For males, only current smokers have significantly higher odds of developing psychological distress 8 years later (adjusted odds ratio (aOR) = 1.76, 95 % CI 1.24—2.48).

Regardless of location, ETS exposure always leads to worse wellbeing and higher likelihood of psychological distress (Table 4). Males and females exhibited different patterns of responses to ETS exposure. For females, exposure at all locations strongly correlated with worse physical and mental wellbeing. In contrast, among males, only ETS exposure at transport stations led to a weak association with decreased physical wellbeing; all other locations showed no statistically significant link.

**Table 4:** Eight year longitudinal wellbeing and psychological distress for passive smokers by location of ETS exposure^a^

Location of ETS exposure		Health outcomes					
		Wellbeing scores (SF-8)^b^				Psychological Distress (Kessler 6)^c^	
		Physical Component		Mental Component			
		aMD^d^	95 % CI	aMD^d^	95 % CI	aOR^e^	95 % CI
Overall	Home	−0.48***	−0.67 to −0.28	−0.59***	−0.81 to −0.37	1.21***	1.14 to 1.29
	Recreational places	−0.28**	−0.48 to −0.07	−0.52***	−0.75 to −0.29	1.23***	1.15 to 1.32
	Work	−0.45***	−0.63 to −0.29	−0.58***	−0.77 to −0.38	1.23***	1.16 to 1.30
	Transport station	−0.37***	−0.54 to −0.21	−0.35***	−0.53 to −0.16	1.10***	1.04 to 1.17
Male	Home	−0.20	−0.62 to 0.23	−0.54*	−1.02 to −0.07	1.23*	1.06 to 1.42
	Recreational places	−0.16	−0.50 to 0.19	−0.58**	−0.96 to −0.19	1.37***	1.21 to 1.54
	Work	−0.23	−0.54 to 0.07	−0.62***	−0.96 to −0.27	1.13*	1.01 to 1.26
	Transport station	−0.30*	−0.60 to 0.00	−0.24	−0.57 to 0.01	1.15*	1.04 to 1.29
Female	Home	−0.54***	−0.76 to −0.31	−0.60***	−0.85 to −0.34	1.21***	1.12 to 1.30
	Recreational places	−0.34**	−0.59 to −0.09	−0.49**	−0.78 to −0.21	1.18**	1.08 to 1.27
	Work	−0.55***	−0.75 to −0.34	−0.55***	−0.79 to −0.32	1.27***	1.18 to 1.36
	Transport station	−0.41***	−0.61 to −0.21	−0.40***	−0.62 to −0.17	1.09*	1.02 to 1.16

*p < 0.05 **p < 0.01 ***p < 0.001

^a^adjusted for demographic attributes (age group, sex, marital status, urbanisation), SES (assets, income, education), and other factors (drinking, number of health conditions, body sizes)

^b^multiple linear regression for SF-8 physical and mental component scores (PCS & MCS)

^c^binary logistic regression for psychological distress (K6 14–30) vs non distress (K6 6–13)

^d^adjusted mean difference

^e^adjusted odds ratio

For females, ETS at home was the principal source of low MCS scores. For males, exposure to ETS at workplaces led to the largest fall in MCS score. For both sexes, exposure to ETS at all sources increased the odds of reporting psychological distress. Among males, exposure at recreational areas showed the strongest association with reporting psychological distress (aOR = 1.37, 95 % CI 1.21—1.54). Meanwhile, for females, highest likelihood of psychological distress were found amongst those reporting exposure to ETS at the workplace (aOR = 1.27, 95 % CI 1.18—1.36) or at home (aOR = 1.21, 95 % CI 1.12—1.30).

## Discussion

We used the TCS, a large cohort of adults in Thailand, to investigate environmental tobacco smoke exposure and associated disparities in wellbeing and psychological distress. A high proportion of Thais, especially females, were passive smokers at home and at public transport stations. In contrast, for men passive smoking was more common at the workplace and during recreation. There was significant longitudinal association between baseline smoking status and health and wellbeing outcomes 8 years later. There were disparities in health by levels of smoke exposure with progressive worsening of the health outcomes among current, former, and passive smokers compared to the non-exposed group.

The impact of the enforcement of smoke-free legislations in Western societies has already shown benefits in reducing adverse health outcomes such as coronary heart diseases and respiratory health [23–25]; but longitudinal monitoring over time is needed for public health policy and intervention to continue minimizing the environmental smoke exposure. As well, although the ban of public smoking in confined spaces has already been introduced for over a decade and was quite successful in Western countries [26], the enforcement impact was relatively low elsewhere [27–29]. Smoking still has high prevalence in developing countries, differentially affecting the most vulnerable sub-groups who are economically worse off.

Investigation of the impact of environmental smoke exposure should not only focus on banning in public areas but also the impacts at home [30, 31]. With a public ban of smoking, a study in Bangladesh has shown that a relatively large number of adults were still exposed to second-hand smoking at home [32]. So promotion of policy on smoke-free homes will improve health, in particular among the low socioeconomic groups. In Thailand, a recent study revealed that urban women were exposed to passive smoke both at home (from spouse) and the workplace and they were almost four times at a higher risk of breast cancers than non-exposed groups [33]. Our study showed similar results that passive smoking was still common among Thais and we further provided supporting evidence on longitudinal impacts of environmental smoke exposure on health and wellbeing.

Our current study adds to limited evidence on environmental exposure and health in transitional economies. The strength of the study lies in its large prospective longitudinal data with comprehensive baseline demographic, socioeconomic, and health background information and subsequently follow-up in 2005, 2009, and most recently in 2013.

We acknowledge some limitations of our study. First, in each 4-year follow-up wave approximately 70 % of the cohort members were reached. Cohort attrition is common among longitudinal studies and we further investigated that this was generally associated with young mobile cohort members; however, we did not find these dropouts have substantive impacts on smoke exposure status. Second, our cohort members were adult open-university students residing nationwide, who shared similar demographic and geographic attributes with the Thai population; however they had relatively higher education which could lower their smoking prevalence compared to general Thais. Third, the environmental smoke exposure information was only collected at baseline in 2005 which was before the introduction of a broad ban of public smoking in Thailand [34]. We know there are enforcement problems with ETS control and our longitudinal data still captured differentials in health and wellbeing several years after the banning in hotel lobbies, pubs and bars [11, 12].

Future follow-up of the cohort will provide valuable insight into the long-term health impacts by smoking status. If Thailand actively promotes a smoke-free indoor environment, especially at home, the ETS effect reported here will attenuate and the population will benefit.

## Conclusions

We add to current knowledge on the adverse impact of smoking on disparities in both physical and mental health. Active smoking affected current and former smokers but the adverse impact on health and wellbeing amongst passive smokers was also apparent even after nearly a decade of follow-up. The government effort on tobacco control should go beyond minimizing environmental smoke exposure in public. The promotion of smoking cessation programs at home could also potentially reduce disparities in health and wellbeing in middle and lower income settings such as Thailand.

## Abbreviations

aMD: adjusted mean difference.
aOR: adjusted odds ratio
ETS: environmental tobacco smoke
K6: Kessler 6
MCS: Mental Component Summary
MOS SF-8: Medical Outcome Short Form-8
PCS: Physical Component Summary
SES: socioeconomic status
TCS: Thai Cohort Study
